# Jehovah’s Witness patients presenting with ruptured ectopic pregnancies: two case reports

**DOI:** 10.1186/1752-1947-8-312

**Published:** 2014-09-19

**Authors:** Niamh C Murphy, Niamh E Hayes, Fionnuala B Ní Ainle, Karen M Flood

**Affiliations:** 1 Department of Obstetrics and Gynaecology, Rotunda Hospital, Dublin, Ireland; 2 Department of Anaesthesia, Rotunda Hospital, Dublin, Ireland; 3 Department of Haematology, Rotunda Hospital, Dublin, Ireland

**Keywords:** Cell salvage, Ectopic pregnancy, Jehovah’s Witnesses, Surgery

## Abstract

**Introduction:**

The management of emergencies in Jehovah’s Witnesses presents several challenges to obstetricians and gynaecologists. We present two cases of ectopic pregnancies in Jehovah’s Witnesses recently managed in our institution. This is the first case review series of its kind that we could identify. We feel it is of clinical importance for all physicians caring for Jehovah’s Witnesses.

**Case presentation:**

The first patient was a 28-year-old Caucasian Irish woman who presented in a state of collapse and a ruptured ectopic pregnancy was suspected. She refused treatment and took her own discharge against the advice of senior hospital staff. She re-presented to our Emergency Room 6 hours later in hypovolaemic shock. She ultimately consented to blood products including plasma and platelets and underwent laparoscopic left-sided salpingectomy. This consent was queried postoperatively by her next-of-kin but the validity of her consent was clarified by the hospital legal team.

The second patient was a 35-year-old Nigerian woman who presented to our Emergency Room with a 2-week history of intermittent vaginal bleeding and abdominal pain with a haemoglobin of 5.4g/dL. An ectopic pregnancy was diagnosed following assessment. She refused all blood products and underwent right-sided salpingectomy. Intravenous tranexamic acid was administered and cell salvage employed intraoperatively.

**Conclusions:**

We feel that this case review series emphasises the importance of appropriate management of Jehovah’s Witnesses in our units. In both of the above cases, these women were in potentially life-threatening situations. Advances in haematology and pharmaceutical therapy contributed to their survival. We welcome these advances in the treatment of this patient population.

## Introduction

Jehovah’s Witnesses are members of a group who refuse blood products on religious grounds based on a doctrine introduced in 1945. They believe that the Bible prohibits ingesting blood and this is based on an interpretation of scripture that differs from most Christian denominations. Furthermore, Jehovah’s Witnesses who accept blood are considered to have dissociated themselves from the religion [1] and can subsequently be shunned [2].

Several unique issues arise when health professionals provide obstetric care to Jehovah’s Witnesses. It has been shown that if such women have an obstetric haemorrhage, they are at a 44-fold increased risk of maternal mortality [3].

This necessitates the need for alternative management and therapeutic options.

## Case presentation

Patient 1 was a 28-year-old Caucasian Irish woman in her first pregnancy. She presented to our Emergency Room (ER) in July 2012 with vaginal bleeding. Her last menstrual period (LMP) was 5 weeks and 3 days previously. An ultrasound scan showed an empty uterus and she was referred to our Early Pregnancy Unit for further assessment. A subsequent transvaginal ultrasound showed a left-sided adnexal mass with a background of serial serum beta subunit of human chorionic gonadotropin (beta-HCG) levels that were plateauing (94 to 108IU/L).

She initially refused any intervention and discharged herself against medical advice. She stated that she did not want any medical treatment and was advised fully of the risks of declining same, including death or severe maternal morbidity, by senior staff members. Our unit takes the issue of very ill patients self-discharging very seriously, given the obvious potential risks to their lives or well-being. On this occasion, we followed every protocol within the hospital, including having the most senior staff available to meet with her to advise her fully on the gravity of the situation. She was, however, found to be of sound mind and thus we could not force her to stay against her will. She left in the care of her partner and signed a form advising that she was taking her discharge against medical advice. Her general practitioner was also contacted advising them of the situation; her general practitioner assured our team that they would contact her directly themselves to attempt to persuade her to return.

Subsequently she presented to the ER 6 hours later in a state of collapse with hypotension and tachycardia. Urgent bloods were procured and her haemoglobin level was found to be 90g/L. She was reviewed by senior obstetric and anaesthetic consultants and consented to theatre. She was advised that this could be a life-threatening situation but she refused to accept red blood cell transfusion intraoperatively based on her religious beliefs. She consented to platelets and plasma transfusions. She underwent laparoscopic right-sided salpingectomy. Intraoperatively, a haemoperitoneum of 2 to 3 litres was noted. The use of cell salvage was also implemented to minimise blood loss.

Postoperatively, her partner advised hospital staff that she was not in a position to consent to platelets or plasma given her religious beliefs. He furthermore produced written directives, which he stated had previously been signed by the patient declining any blood products. The hospital legal team were contacted and advised as follows: “Physicians are not bound by written directives which they have not seen themselves. The initial verbal consent is valid and supercedes the written directive signed even if same is produced by the partner”. She was stable postoperatively and warranted no further intervention.

Patient 2 was a 35-year-old Nigerian woman in her second pregnancy. She had a previous normal delivery. She presented to the ER in January 2013 with PV spotting. Her serum beta-HCG was elevated (3900IU/L). Her LMP was 5 weeks previously. No intrauterine pregnancy was diagnosed and she was referred to our Early Pregnancy Unit for evaluation of pregnancy of unknown location. An ultrasound scan showed an empty uterus with free fluid and right adnexal mass. Haemoglobin sent at this time was processed as 57g/L and she was contacted to attend the ER for urgent assessment. Her vital signs were stable initially but she later became hypotensive and tachycardic.

She declined all blood products. She was again counselled by senior hospital obstetric and anaesthetic consultants about the gravity of the situation. She spoke at length with her pastor before finally consenting to surgery. Intraoperatively, she was given 1g tranexamic acid intravenously. The ectopic was of significant size (65×65×21mm) and 1.5 litres haemoperitoneum was noted. Cell salvage was again used to minimise blood loss. Ischaemic electrocardiogram changes were noted postoperatively including ST depression consistent with hypoxic damage. Her postoperative haemoglobin was 5.2 and she was discharged 48 hours later with advice to take Galfer (ferrous fumarate) twice daily.

## Conclusions

It is of vital importance that medical staff caring for Jehovah’s Witnesses be familiar with all management options. In the cases above, the additional input of cell salvage and the work of a multidisciplinary team were crucial and potentially life-saving.

All Jehovah’s Witness patients should undergo assessment and discussion with senior consultant haematology, obstetric and anaesthetic staff [4]. Options available as alternatives to red blood cells include prevention of anaemia to maintain haematocrit above 40% [5]. Tranexamic acid was used for patient 2, which is a commonly used medication for patients experiencing bleeding intraoperatively in our unit. Anti-embolic deterrent stockings were used to prevent thrombosis. For elective cases, erythropoietin may also be considered for a patient with a haematocrit of less than 40%. Erythropoietin stimulates the bone marrow to maximise red blood cell production. Not all Jehovah’s Witnesses will accept this medication because the drug is packaged with 2.5mL of albumin per dose [6].

Autologous blood donation involves optimising the patient’s haematocrit with oral iron supplementation (or erythropoietin if acceptable) and then having her donate her own blood at least 72 hours (but, ideally, 2 weeks) before elective caesarean delivery or estimated date of delivery [7].

Aside from allogeneic blood or blood products, other options should also be discussed. In our unit, staff are trained in the correct usage of cell salvage systems and same can be used in an emergency setting. Cell salvage systems are also an option in an elective setting. This can be employed as a form of intraoperative autologous blood donation. “Cell saver” systems allow for free blood in the abdomen to be aspirated, filtered, and then reinfused into the patient perioperatively as an intraoperative autologous transfusion [8]. This is of significant importance given its life-saving capabilities and the fact that it is generally acceptable to members of the Jehovah’s Witness community given that it does not involve transfusion of blood products. Consent in this regard is crucial.

In our first case above, the issue of informed consent was raised and the hospital legal team were consulted. The issue of whether or not she may have been in a position to give informed consent was raised given her potential hypoxia and pain, both of which could contribute as extraneous factors to the ability of giving informed consent. It was felt overall that she was of sound mind at the time and thus her consent was considered to be both informed and valid.

Any Jehovah’s Witness patient in a life-threatening situation must be reviewed by the most senior available anaesthetic, obstetric and haematological staff. They must be made aware of the gravity of the situation and hospital legal experts should be consulted where necessary.

All options should be discussed and a management plan should be clearly documented.

Our focus was on obstetric and gynaecological care of Jehovah’s Witnesses. However, we feel that this discussion could also be relevant in other areas of medicine and surgery. We feel that advances in haematological and pharmaceutical treatments can aid all members of this patient population.

## Consent

Written informed consent was obtained from the patients for publication of this case report and accompanying images. A copy of the written consent is available for review by the Editor-in-Chief of this journal.

## Competing interests

The authors declare that they have no competing interests.

## Authors’ contributions

NM reviewed the charts and provided the literature review. NH contributed to anaesthetic and cell salvage knowledge. FNA advised on haematological advances. KF was involved in the initial surgeries and provided overall lead. All authors read and approved the final manuscript.

## References

[B1] Osamu M Bioethical aspects of the recent changes in the policy of refusal of blood by Jehovah’s Witnesses Br Med J 2001 322 37 10.1136/bmj.322.7277.37 PMC1119307 11141155

[B2] Jehovah’s Witnesses Public Affairs Office Press Release 2000

[B3] Singla AK Lapinski RH Berkowitz RL Saphier CJ Are women who are Jehovah’s Witnesses at risk of maternal death? Am J ObstetGynecol 2001 185 893 895 10.1067/mob.2001.117357 11641673

[B4] Obstetrical Guidelines for Jehovah's Witnesses Patients 1996 New York: The Mount Sinai School of Medicine, The Department of Obstetrics, Gynecology, and Reproductive Sciences

[B5] Gyamfi C Yasin SY Preparation for an elective surgical procedure in a Jehovah’s Witness: a review of the treatments and alternatives for anemia Prim Care Update Ob Gyns 2000 7 266 268 10.1016/S1068-607X(00)00057-3 11077241

[B6] Gyamfi C Gyamfi MM Berkowitz RL Ethical and medicolegal considerations in the obstetric care of a Jehovah’s Witness Obstetr Gynaecol 2003 102 1 173 180 10.1016/S0029-7844(03)00236-9 12850626

[B7] Yamada AH Lieskovsky G Skinner DG Shulman I Groshen S Chen S Impact of autologous blood transfusion on patients undergoing radical prostatectomy using hypotensive anesthesia J Urol 1993 149 73 76 8417219 10.1016/s0022-5347(17)36002-0

[B8] Desmond MJ Thomas MJG Gillon J Fox MA Perioperative red cell salvage Transfusion 1996 36 644 651 10.1046/j.1537-2995.1996.36796323065.x 8701462

